# Quantitative myocardial perfusion imaging using a step arterial-input function

**DOI:** 10.1186/1532-429X-18-S1-O11

**Published:** 2016-01-27

**Authors:** Richard B Thompson, Justin Grenier, Emer Sonnex, Richard Coulden

**Affiliations:** 1 grid.17089.37Biomedical Engineering, University of Alberta, Edmonton, AB Canada; 2 grid.17089.37Radiology and Diagnostic Imaging, University of Alberta, Edmonton, AB Canada

## Background

**Figure 1:**
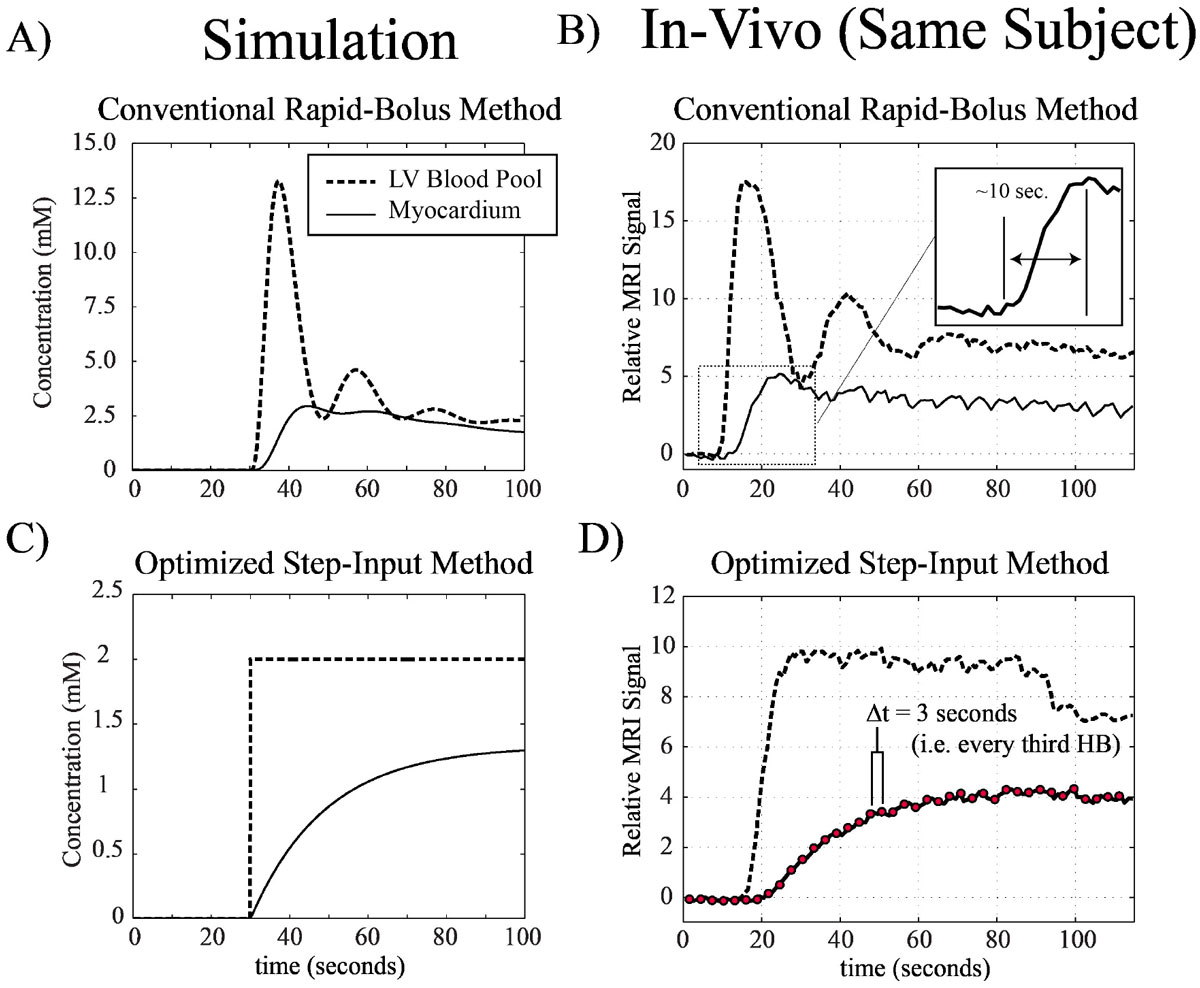
**A)** Simulated arterial input and tissue contrast agent concentration (based on 5 ml/s bolus injection and 1 ml/g/min perfusion). **B)** In-vivo blood (LV pool) and myocardial signal (normalized to baseline) for a bolus injection (XX ml at 5 ml/s) in a healthy control. **C)** Myocardial tissue response for an idealized step-input for 1 ml/g/min perfusion. **D)** In-vivo blood (LV pool) and myocardial signal (normalized to baseline) for an optimized pseudo-step-input protocol (same subject as **B**).

Modern MRI myocardial perfusion protocols use rapid venous bolus injections, typically 3-5 ml/s of 5-15 ml of agent over a few seconds. The resulting arterial input functions are rapidly varying with high agent concentrations (Fig. 1A and 1B) and thus typically require high temporal resolution acquisitions (~1 sec), custom pulse sequences and complex processing methods for perfusion quantification. A new myocardial perfusion approach, based on a pseudo step arterial-input function (Magn Reson Med. 2005 Aug;54(2):289-98), is introduced that offers simplified and lower concentration input functions, simplified quantitative data processing and reduced demands for high temporal resolution.

## Methods

Numerical simulations of whole body vascular systems were used to design optimized venous injection protocols for the generation of step-input-like arterial-input functions targeting the idealized step-input function show in Fig. 1C. A two-compartment numerical model was used to estimate myocardial contrast agent concentration dynamics for conventional (bolus) and step-input protocols.

In-vivo experiments were performed on a Siemens Aera 1.5T (Siemens Healthcare, Erlangen, Germany). ECG-gated saturation-recovery (TS=100 ms) bSFFP images were acquired for 120 heartbeats (1 image/beat, diastasis). Matrix size 224 × 136, rate 2 GRAPPA, 8 mm slice, 1.03 ms TE, 2.5 ms TR, 70° flip. All contrast injections were single dose (0.1 mmol/kg) of Magnevist (Bayer). In-vivo data was acquired in 3 healthy controls and 3 CAD patients, all ~90 days post MI (LVEF = 45%-66%, 61-92 kg). Blood/tissue signal intensities were converted to contrast agent concentrations using a Bloch equation look-up-table approach and myocardial perfusion was estimated with an exponential deconvolution approach.

## Results

**Figure 2:**
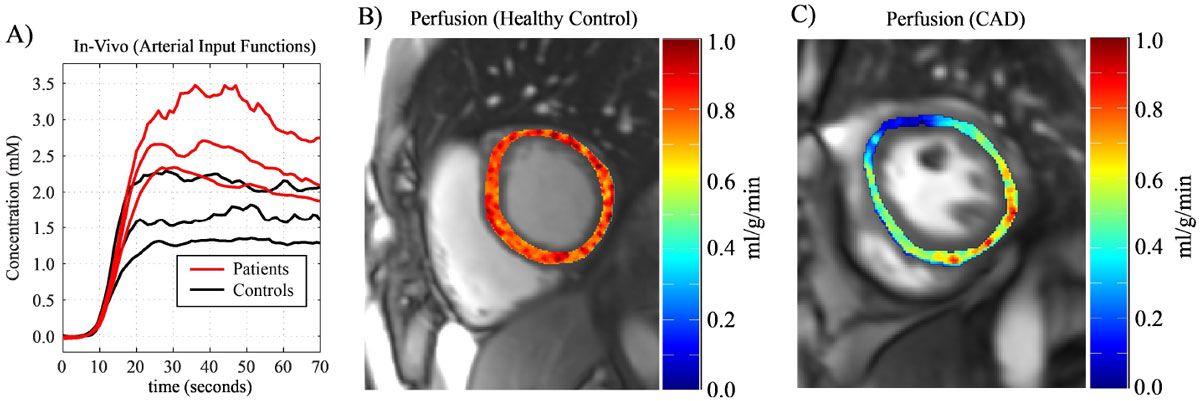
**A) Left ventricular arterial input functions for the 6 study subjects using the optimized step input venous injection protocol**. Sample quantitative perfusion images for a healthy control subject and a patient with coronary artery disease (CAD) are shown in **B)** and **C)**, respectively.

Optimized venous injection protocols comprised decaying injection rates over ~1 min. with contrast agent dilution to ~60 ml (same protocol for all subjects). Sample blood and tissue time-intensity curves (normalized to baseline) in a healthy subject are shown in Fig. 1B and 1D, for a standard rapid bolus and an optimized step-input injection protocol. Fig. 2A shows arterial inputs for all subjects, and a sample perfusion map in a healthy control and patient are shown Fig. 2B and 2C.

## Conclusions

A generalizable injection protocol can generate a pseudo arterial step-input function for a range of subject sizes and heart function, offering several advantages over conventional bolus injections: slower tissue dynamics enable multi-slice imaging with single-slice per heart-beat acquisitions, lower concentrations mitigate T_2_* and T_1_ saturation effects and long injection duration avoids recirculation effects. The conventional short tissue "dynamic" window (~10 seconds, Fig. 1B inset) reflects complex bolus injection dynamics; the pseudo-step arterial input reveals a longer window (~60 seconds, Fig. 1D) over which the contrast agent redistributes to the tissue via perfusion (as predicted with compartmental modeling in Fig. 1C).

